# Pre-pregnancy predictors of hypertension in pregnancy among Aboriginal and Torres Strait Islander women in north Queensland, Australia; a prospective cohort study

**DOI:** 10.1186/1471-2458-13-138

**Published:** 2013-02-15

**Authors:** Sandra K Campbell, John Lynch, Adrian Esterman, Robyn McDermott

**Affiliations:** 1 School of Health Sciences, Division of Health Sciences, University of South Australia, City East Campus North Terrace (P5-21), GPO Box 2471, 5001, Adelaide, SA, Australia; 2 Discipline of Public Health, University of Adelaide, Adelaide, SA, Australia; 3 School of Nursing and Midwifery, Division of Health Sciences, University of South Australia, Adelaide, SA, Australia; 4 Sansom Institute, Division of Health Sciences, University of South Australia, Adelaide, SA, Australia

**Keywords:** Indigenous, Pre-pregnancy, Hypertension, Preeclampsia

## Abstract

**Background:**

Compared to other Australian women, Indigenous women are frequently at greater risk for hypertensive disorders of pregnancy. We examined pre-pregnancy factors that may predict hypertension in pregnancy in a cohort of Aboriginal and Torres Strait Islander women in north Queensland.

**Methods:**

Data on a cohort of 1009 Indigenous women of childbearing age (15–44 years) who participated in a 1998–2000 health screening program in north Queensland were combined with 1998–2008 Queensland hospitalisations data using probabilistic data linkage. Data on the women in the cohort who were hospitalised for birth (n = 220) were further combined with Queensland perinatal data which identified those diagnosed with hypertension in pregnancy*.*

**Results:**

Of 220 women who gave birth, 22 had hypertension in the pregnancy after their health check. The mean age of women with and without hypertension was similar (23.7 years and 23.9 years respectively) however Aboriginal women were more affected compared to Torres Strait Islanders. Pre-pregnancy adiposity and elevated blood pressure at the health screening program were predictors of a pregnancy affected by hypertension. After adjusting for age and ethnicity, each 1 cm increase in waist circumference showed a 4% increased risk for hypertension in pregnancy (PR 1.04; 95% CI; 1.02-1.06); each 1 point increase in BMI showed a 9% adjusted increase in risk (1.09; 1.04-1.14). For each 1 mmHg increase in baseline systolic blood pressure there was an age and ethnicity adjusted 6% increase in risk and each 1 mmHg increase in diastolic blood pressure showed a 7% increase in risk (1.06; 1.03-1.09 and 1.07; 1.03-1.11 respectively). Among those free of diabetes at baseline, the presence of the metabolic syndrome (International Diabetes Federation criteria) predicted over a three-fold increase in age-ethnicity-adjusted risk (3.5; 1.50-8.17).

**Conclusions:**

Pre-pregnancy adiposity and features of the metabolic syndrome among these young Aboriginal and Torres Strait Islander women track strongly to increased risk of hypertension in pregnancy with associated risks to the health of babies.

## Background

Hypertensive disorders complicate 5-10% of pregnancies and contribute greatly to maternal and fetal morbidity and mortality throughout the world [1-3]. Population-based Australian studies undertaken in Victoria [4] and South Australia [5] have shown a prevalence of 9.8% and 9.2% respectively, however, Australian Indigenous women have been reported as having 66% greater risk for pregnancy-induced hypertension when compared with non-Indigenous women [6]. Australian Aboriginal and Torres Strait Islander women are also at comparatively higher risk for developing chronic hypertension and cardiovascular disease at a younger age than other Australian women [7,8]. Poor cardiovascular health prior to pregnancy modulates maternal preeclamptic syndrome so that frequency and severity of hypertensive disease in pregnancy are increased [9]. Women with chronic hypertension may have uneventful pregnancies when their hypertension remains mild to moderate, however, overall outcomes are poorer than for normotensive women and they are at at greater risk of developing preeclampsia (≥20%) [10]. Preeclampsia occurs in about 3-5% of pregnancies [11] and is the hypertensive condition whether it occurs alone or superimposed on chronic hypertension most often associated with maternal and fetal complications including fatalities [1]. The disorder is an early marker not only for future maternal cardiovascular and metabolic disease, [1,11,12] but also confers long-term risk for cardiovascular disease among infants [2]. Hypertension during pregnancy has been identified as a predictor of poor birth outcomes in Aboriginal and Torres Strait Islander women in north Queensland [13]. However, it remains unclear why this group of women have an increased risk for hypertension in pregnancy.

Pre-pregnancy overweight and obesity have been identified as risk factors for hypertensive disorders of pregnancy [14,15]. In the United States, obesity is the leading risk factor for preeclampsia; it confers a three-fold increased risk and is present in 30% of cases [16]. Now a major epidemic in developed countries including Australia, obesity is impacting on the health and well-being of Indigenous Australians. With the change from traditional carbohydrate based diets to energy dense high fat diets, combined with lower levels of physical activity [17], rates of obesity among Aboriginal and Torres Strait Islander women of childbearing age have escalated [18]. We examined pre-pregnancy factors that may predict any hypertension in pregnancy in a cohort of Aboriginal and Torres Strait Islander women in north Queensland. We hypothesised that abnormal pre-pregnancy blood pressure levels and maternal adiposity would increase the risk of hypertensive disorders during pregnancy.

## Methods

### Study population

The Aboriginal and Torres Strait Islander women in this study were participants in the March 1998-December 2000 Well Person’s Health Check (WPHC) cross-sectional health survey [19], which included 26 rural and remote Indigenous communities in the Bowen, Cairns, Cape York, Torres Strait and Mount Isa Health service districts. All adults were invited to participate through printed media, local radio and word of mouth via the local health service, community council and community groups. The survey was attended by 2862 Indigenous people aged 15 years and over, giving an overall participation rate of 44.5% (according to local census data) [20]; 51.7% were female. Participants overall, were not different demographically from the age and sex distribution of the Indigenous population who did not attend, based on census data. A subset of women from the original survey data were selected for this study because the WPHC occurred when they were aged between 15–44 years (a generally accepted range of childbearing age).

### Data sources

Three datasets were linked to bring together information on maternal pre-pregnancy factors (anthropometric, biochemical and lifestyle) gathered at the WPHC, and the presence or absence of a hypertensive disorder during the next pregnancy following this health check. First, records of Aboriginal and Torres Strait Islander women of childbearing age who attended the WPHC (details of the survey are outlined below) were linked to Queensland hospital Unit Registration numbers by probabilistic matching of name, date of birth and residential address. The second dataset contained hospitalisations data (including International Classification of Disease (ICD) 9 and ICD 10 codes and adjacent diagnosis-related groups and descriptions) identified those women who were hospitalised for pregnancy-related conditions after the date of their WPHC up to the study censor date on 30 March 2008. To idenify women who had a hypertensive disorder of pregnancy after the WPHC, a third dataset provided by Statistical Output, Health Statistics Centre, Queensland Health was added. The third dataset included perinatal data specific to those women who had been hospitalised for birth of an infant of at least 20 weeks completed gestation or a birthweight >400 g when gestation was unknown [21]. When a woman was hospitalised for more than one birth during the follow-up period, only perinatal data for the birth nearest the health check was requested. Women who were likely to have been pregnant at the time of their health check were excluded based on the date of their health check, the date of their delivery and the estimated gestation of the pregnancy at the time of birth.

**Figure 1:**
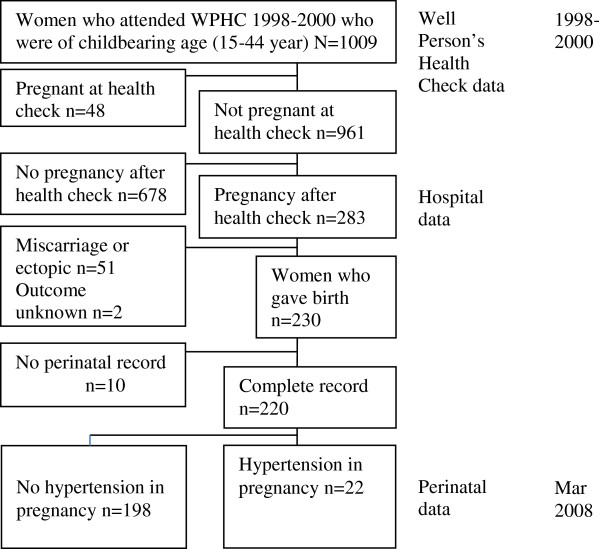
Flow chart including datasets linked (right) and timeline (far right).

ICD codes within the perinatal data identified women who had hypertensive conditions (ICD O10-O16) during their pregnancy (Figure 1). The outcome measure “hypertension in pregnancy” in this study applies to women who were recorded in the perinatal dataset as receiving a diagnosis of one of the forms of the disorder; pre-existing hypertension (ICD O10), pre-existing hypertension with superimposed preeclampsia-eclampsia (ICD O11), gestational hypertension without proteinuria (ICD 013) and preeclampsia-eclampsia (ICD O14 and O15).

### Well person’s health check

A standardised proforma was used for data collection in the baseline WPHC survey. Body measurements and specimen collection were performed by a multidisciplinary team of trained staff from the Cairns Tropical Public Health Unit (Queensland Health) and local health staff in each communtiy. Face-to-face interviews followed a structured questionnaire. Fasting venous blood samples were collected by a medical officer, registered nurse or trained phlebotomist [19].

#### Anthropometric measures

Participants were invited to attend in the early morning following at least an 8 h fast. They were weighed in light clothing to the nearest 0.1 kg on digital electronic scales and height was measured to the nearest centimetre. Body mass index (BMI) was calculated as weight (kg) divided by height squared (m^2^). Waist circumference was measured to the nearest centimetre. Anthropometric cut-offs were set according to World Health Organisation criteria [22].

#### Blood pressure

Seated blood pressure was recorded three times over approximately 10 min with an automated blood pressure monitor, and mean systolic and diastolic measurements were calculated.

#### Self-reported behavioural factors

Fruit and vegetable intake was self-reported by 24-h recall and 7-day recall was used to assess the duration and intensity of physical activity. The participants were categorised as having adequate physical activity if they reported a minimum of 30 min of moderate to vigorous exercise on at least 5 days in the week. Current smoking status was self-reported. Participants who drank alcohol self-reported the types and amounts of alcohol consumed in the week before the health check; they were then categorised as being safe drinkers, non-drinkers or harmful (“risky”) drinkers according to Australian National Health and Medical Research Council guidelines (2001). Risky drinking for women was defined as more than two standard drinks per day or more than four on any single occasion. The physical activity, smoking and alcohol intake measures have been widely used [23] and the 24-h recall dietary questionnaire and red cell folate measure have been validated against other measures of micronutrient intake and the quality of the diet with respect to fruit and vegetables [24,25].

#### Biochemical measures

Early-morning fasting venous blood samples were collected in ethylenediamine tetra-acetic acid vacuum tubes and clotted serum separator vacuum tubes (spun for 10 min at 1000 RCF in a swinging bucket centrifuge and separated within 1 h). Samples were stored in 4-8°C and transported for analysis within 24 hours. The biochemical measurements included glucose, triglycerides, total cholesterol, high density lipoprotein cholesterol, γ-glutamyl transferase and red cell folate. Glucose and lipids were measured using photometric enzyme endpoint assay with Cobas Integra 700/400 (Roche Diagnostic, Switzerland). γ -Glutamyl transferase was measured by a kinetic photometric procedure with Cobas Integra 800 (Roche Diagnostic, Switzerland). An elevated level was defined as ≥ 50 U/L and < 50 U/L as normal, according to criteria established by Queensland Health Pathology Service (http://www.health.qld.gov.au/qhcss/qhps/default.asp). Red cell folate was measured with the Bayer Advia Centaur automated immunoassay system (Bayer, Australia) by the Queensland Health Pathology Service in Brisbane. The reference range for this assay was 295–1800 nmol/L.

Urine specimens collected in sterile 50-ml containers were either the first morning or at least 2 h after the most recent void. If protein was detected by dipstick urinalysis (Combur-test, Roche) or if the participant was known to have diabetes, hypertension or obesity (BMI >30 kg/m^2^), the albumin: creatinine ratio was measured by immunoassay in grams per mole. Microalbuminuria was defined as an albumin:creatinine ratio of 3.4-34 g/mol, and macroalbuminuria as >34 g/mol [26]. From May 1999 to the end of the project, the albumin:creatinine ratio was measured in all urine specimens.

#### Metabolic syndrome

The metabolic syndrome in this study is defined according to the International Diabetes Federation criteria as comprising waist circumference ≥ 80 cm plus two or more of the following: raised triglycerides (≥ 1.7 mmol/L), reduced high-density lipoprotein (< 1.29 mmol/L), raised blood pressure (systolic ≥ 130 mm Hg or diastolic ≥ 85 mm Hg) and raised plasma glucose (≥ 5.6 mmol/L) (http://www.idf.org/metabolic-syndrome).

### Ethics

Ethics approval for the WPHC was provided by the Cairns Base Hospital Ethics Committee in March 1998. The participants provided consent to have their Queensland Health medical records reviewed and linked to their WPHC records. The current data linkage study was approved by the University of South Australia and Cairns Base Hospital Human Research Ethics Committees (June 2009 and September 2009) with support from the peak health councils representing Indigenous people in the region: Apunipima and the Torres Strait Islander and Northern Peninsula Area Health Councils.

### Statistical analysis

The data were analysed in a generalised linear model (Poisson distribution) with robust variance estimates to calculate prevalence ratios (PRs) and 95% confidence intervals (CIs) with 2 sided p-values of 0.05 for baseline characteristics associated with hypertension during the first pregnancy after their health check. Data are presented in both unadjusted and adjusted analyses. An *a priori* decision was taken to adjust the model for age because hypertension is commonly associated with increasing age and, because it was expected that Aboriginal and Torres Strait Islander women would differ in their anthropometic and metabolic characteristics, ethnicity was included in the adjusted model. Further adjustment for months from participation in the baseline survey to time of admission for birth was not associated with the outcome and was not retained. Fruit and vegetable intake was excluded from the analyses due to very small cell sizes. The analysis was conducted with STATA 11 (STATAcorp, College Station, Texas, USA).

## Results

### Pre-pregnancy characteristics at the baseline health check

At the baseline WHPC, there were 1009 women of childbearing age (15–44 years) in the study sample. We excluded 48 women because they were pregnant at the time of their health check. Of the remaining 961 women, 61.2% identified as Aboriginal, 30.7% as Torres Strait Islander, and 8.1% identified as both Torres Strait Islander and Aboriginal. Characteristics of the cohort (demographics, waist circumferences BMIs, smoking status, alcohol use, fruit and vegetable intake, physical activity, blood pressure, blood lipids, red cell folate, fasting glucose, kidney and liver function tests, prevalences of metabolic syndrome and diabetes) are reported in detail elsewhere [20]. Abdominal obesity (waist circumference ≥ 88 cm) was found in 60.5% of women, and 37.1% had a BMI > 30. Adequate fruit and vegetable intake and physical activity were low (1.5 and 21% respectively), and self-reported rates of tobacco smoking and risky drinking in the week before the baseline health check were high (60.8% and 41.9%, respectively).

### Prevalence of hypertension in pregnancy

Figure 1 shows that 283 women were hospitalised for a pregnancy-related condition in the time between their WPHC in 1998–2000 and March 2008; 51 pregnancies ended in miscarriage or because of an ectopic pregnancy, and two pregnancy outcomes were unknown. In the time up to the censor date, 230 women were hospitalised for birth. Ten of these women were excluded because perinatal records were not matched. Of the remaining 220 women (21.4% were primiparous), 9 had pre-existing hypertension at the time of their WPHC. Seven women with normal blood pressure at baseline developed gestational hypertension (new-onset hypertension after mid-pregnancy, in the absence of proteinuria). Seven women (3.2%) developed preeclampsia (hypertension and proteinuria); six cases were in women with normal baseline blood pressure and were described as moderate, and one severe case was superimposed on pre-existing hypertension. Overall, 22 women (10%) had a hypertensive disorder in their first pregnancy following the baseline WPHC.

### Pre-pregnancy characteristics as risk for hypertension in pregnancy: unadjusted analyses

Unadjusted analysis in Table 1 shows that of the 22 women who had hypertension in the first pregnancy after their health check, 72.7% were Aboriginal; the remaining 27.3% of the women identified as Torres Strait Islander or, both Torres Strait Islander and Aboriginal (p =0.157). There was no difference in mean age between the women who did and did not have hypertension in pregnancy (p =0.914). Elevated pre-pregnancy systolic and diastolic blood pressure at the WPHC predicted more than a three-fold risk for hypertension in pregnancy (p = 0.001 and 0.025 respectively). The affected women were more likely to have a higher mean waist circumference (p =0.054) and a mean BMI three points above the women with no hypertension (p = 0.033). More women who had hypertension reported risky drinking (50% versus 38%; p =0.236) and elevated albumin:creatinine ratio and low levels of high density lipoprotein cholesterol were more common (31.6% versus 17.7% and 59.1% versus 43.5% respectively) though not statistically significant (p =0.366 and 0.170 respectively). Non-diabetic women who were diagnosed with hypertension in their pregnancy had more than twice the prevalence of features of the metabolic syndrome at the baseline health check (42.9% versus 19.9%; p = 0.018). Among all the women who had a baby (n = 220), baseline self-reported levels of adequate physical activity were low (19.5%) and smoking rates and risky drinking were high (70% and 38.6% respectively).

**Table 1:** Baseline characteristics of the cohort, by hypertension in pregnancy (n = 220)

**Pre-pregnancy characteristic**	**No hypertension during pregnancy (n = 198)**		**Hypertension during pregnancy (n = 22)**			
	**n**	**% of n or mean (sd)**	**n**	**% of n or mean (sd)**	**Unadjusted PR (95% CI)**	***p *value**
Indigenous group						
Aboriginal	112	56.6	16	72.7	1.00	
Torres Strait Islander^a^	86	43.4	6	27.3	0.52 (0.21-1.28)	0.157
Age years (mean, sd)	198	23.9 (5.4)	22	23.7 (6)	0.99 (0.92-1.07)	0.914
Waist circumference (cm)						
< 80	65	33	5	22.7	1.00	
80-87.9	40	20.3	2	9.1	0.67 (0.13-3.30)	0.619
≥ 88	92	46.7	15	68.2	1.96 (0.74-5.17)	0.372
Waist circumference (mean, sd)	197	88.7 (15.8)	22	95.4 (17.6)	1.02 (1.00-1.04)	0.054
BMI category (kg/m^2^)						
< 18.5	23	11.6	0	0		
18.5-25	74	37.4	7	31.8	1.00	
25-30	48	24.2	6	27.3	1.29 (0.46-3.63)	0.635
≥ 30+	53	26.7	9	40.9	1.68 (0.66-4.26)	0.276
BMI (mean, sd)	198	26.1(7)	22	29 (6.5)	1.05 (1.00-1.09)	0.033
Smoker						
No	57	28.9	8	36.4	1.00	
Yes	140	71.1	14	63.6	0.74 (0.33-3.68)	0.469
Alcohol use						
Safe drinker	56	28.9	4	18.2	1.00	
Non drinker	64	33	7	31.8	1.48 (0.45-4.82)	0.517
Risky drinker	74	38.1	11	50	1.94 (0.65-5.82)	0.236
Enough physical activity						
No	160	80.8	17	77.3	1.00	
Yes	38	19.2	5	22.7	1.23 (0.47-3.11)	0.691
Systolic blood pressure (mmHg)						
≤ 120	142	72.1	8	36.4	1.00	
> 120	55	27.9	14	63.6	3.80 (1.67-8.67)	0.001
Diastolic blood pressure (mmHg)						
≤ 80	190	96.5	19	86.4	1.00	
> 80	7	3.5	3	13.6	3.30 (1.16-9.35)	0.025
Cholesterol (mg/dL)						
≤ 5.5	176	90.3	20	90.9	1.00	
> 5.5 (elevated)	19	9.7	2	9.1	0.93 (0.23-3.73)	0.922
Triglycerides (mmol/L)						
≤ 2	173	89.6	19	86.4	1.00	
> 2 (elevated)	20	10.4	3	13.6	1.31 (0.42-4.12)	0.635
HDL cholesterol (mmol/L)						
> 1	108	56.5	9	40.9	1.00	
≤ 1 (low)	83	43.5	13	59.1	1.76 (0.78-3.95)	0.170
Red cell folate (nmol/L)						
≥ 295	126	65	17	77.3	1.00	
< 295 (low)	68	35	5	22.7	0.58 (0.22-1.50)	0.260
Fasting glucose (mmol/L)						
≤ 5.5	182	93.3	20	90.9	1.00	
> 5.5	13	6.7	2	9.1	1.35 (0.35-5.24)	0.668
Albumin:creatinine ratio (mg/mmol)						
≤ 3.4	121	82.3	13	68.4	1.00	
>3.4	26	17.7	6	31.6	2.25 (0.39-13.05)	0.366
Gamma-glutamyl transferase (U/L)						
≤ 50	183	93.9	20	90.9	1.00	
> 50 (elevated)	12	6.1	2	9.1	1.45 (0.37-5.60)	0.590
Metabolic syndrome^b^						
No	153	80.1	12	57.1	1.00	
Yes	38	19.9	9	42.9	2.63 (1.18-5.88)	0.018

Denominators vary because of missing values.

^a^Women in this group self-identified as Torres Strait Islander or, as both Torres Strait Islander and Aboriginal origin.

^b^Defined by International Diabetes Federation criteria as comprising waist circumference ≥ 80 cm plus two or more of the following: raised triglycerides (≥ 1.7 mmol/L), reduced high-density lipoprotein (< 1.29 mmol/L), raised blood pressure (systolic ≥ 130 mm Hg or diastolic ≥ 85 mm Hg) and raised plasma glucose (≥ 5.6 mmol/L). Women with diabetes excluded.

### Pre-pregnancy characteristics as risk for hypertension in pregnancy: analysis adjusted for age and ethinicity

In a multivariable analysis (Table 2), a strong predictor for having hypertension in pregnancy was elevated baseline blood pressure. For each 1 mmHg increase in pre-pregnancy systolic blood pressure there was an age and ethnicity adjusted 6% increase in risk of hypertension in pregnancy (PR 1.06; 1.03-1.09) and for each 1 mmHg increase in diastolic blood pressure there was a 7% increase in risk (1.07; 95% CI 1.03-1.11). Body fat was also an important risk factor and for each 1 cm increase in waist circumference there was a 4% age and ethnicity adjusted increase in risk of hypertension in pregnancy (1.04;1.02-1.06) and for each 1 point increase in BMI, there was a 9% increase in risk (1.09; 1.04-1.14). The presence of metabolic syndrome at baseline predicted over a three-fold increase in adjusted risk among women free of diabetes at baseline (3.5; 1.50-8.17). For each 1 mg/mmol increase in pre-pregnancy albumin:creatine ratio there was an age and ethnicity adjusted 1% increased risk (1.01; 1.00-1.02). Pre-pregnancy alcohol use was not significantly associated with hypertension in pregnancy however, for each 1 U/L increase in γ-glutamyl transferase, possibly related to non-alcoholic fatty liver disease, there was a 1% increase in adjusted risk (1.01: 1.00-1.02). Pre-pregnancy smoking, physical activity, fasting glucose, red cell folate and blood fats were not statistically significantly associated with hypertension in pregnancy in this study.

**Table 2:** Multivariable analysis for effects of pre-pregnancy factors and hypertension during pregnancy (n = 220)

**Pre-pregnancy characteristic**	**Unit increase**	**Crude PR (95% confidence interval)**	***p*value**	**PR adjusted for age and ethnicity (95% confidence interval)**	***p*value**
Indigenous group					
Aboriginal	-	1.00			
Torres Strait Islander^a^	-	0.52 (0.21-1.28)	0.16		
Age	“1 year”	1.00 (0.92-1.07)	0.91		
Waist circumference	“1 cm”	1.02 (1.00-1.04)	0.05	1.04 (1.02-1.06)	0.001
BMI (kg/m2)	“1 unit”	1.05 (1.00-1.09)	0.03	1.09 (1.04-1.14)	<0.001
Alcohol intake					
Safe drinker	-	1.00		1.00	
Non drinker	-	1.47 (0.45-4.82)	0.52	1.38 (0.43-4.47)	0.59
Risky drinker	-	1.94 (0.64-5.91)	0.24	1.79 (0.58-5.49)	0.31
Non-smoker	Smoker	0.74 (0.33-1.68)	0.47	0.68 (0.30-1.55)	0.36
Enough physical activity	< 30 min/day 5 days per week	0.83 (0.32-2.12)	0.69	0.78 (0.31-1.55)	0.60
Systolic blood pressure	“1 mmHg”	1.05 (1.02-1.07)	<0.001	1.06 (1.03-1.09)	<0.001
Diastolic blood pressure	“1 mmHg”	1.06 (1.03-1.10)	<0.001	1.07 (1.03-1.11)	<0.001
Cholesterol	“1 mmol/L”	1.27 (0.88-1.83)	0.21	1.38 (0.93-2.06)	0.10
Triglycerides	“1 mmol/L”	1.18 (0.96-1.46)	0.12	1.17 (0.92-1.49)	0.20
HDL cholesterol (≤ 1 = low)	“1 mmol/L”	0.74 (0.13-4.22)	0.73	0.61 (0.09-3.82)	0.60
Red cell folate	“1 nmol/L”	1.00 (1.00-1.01)	0.006	1.00 (1.00-1.01)	0.009
Fasting glucose	“1 mmol/L”	1.06 (0.82-1.37)	0.67	1.14 (0.87-1.50)	0.33
Albumin:creatinine ratio	“1 mg/mmol”	1.01 (1.00-1.02)	0.02	1.01 (1.00-1.02)	0.003
Gamma-glutamyl transferase	“1 U/L”	1.01 (1.00-1.02)	0.02	1.01 (1.00-1.02)	0.003
Hyper-triglyceridemic waist^b^ - No	Yes	1.55 (0.40-5.93)	0.53	1.53 (0.39-6.01)	0.54
Metabolic syndrome^c^ - No	Yes	2.63 (1.18-5.88)	0.02	3.50 (1.50-8.17)	0.004

Denominators vary because of missing values.

^a^Women in this group self-identified as Torres Strait Islander or, as both Torres Strait Islander and Aboriginal origin.

^b^Defined as raised triglycerides (> 2 mmol/l and waist circumference ≥ 88 cm).

^c^Defined by International Diabetes Federation criteria as comprising waist circumference.

≥ 80 cm plus two or more of the following: raised triglycerides (≥ 1.17 mmol/l), reduced high-density lipoprotein (< 1.29 mmol/L), raised blood pressure (systolic ≥ 130 mmHg or diastolic ≥ 85 mmHg) and raised plasma glucose (≥ 5.6 mmol/L). Women with diabetes excluded.

## Discussion

Twenty-two of the 220 women who gave birth in the 8–10 years after their health check had hypertension during their pregnancy. Seven of these women (3.2%) were diagnosed with moderate (n = 6) or severe (n = 1) preeclampsia, a rate equivalent to that found among other population groups [11]. The strongest pre-pregnancy predictors of hypertension during pregnancy in this cohort of Aboriginal and Torres Strait Islander women were central adiposity (assessed by waist circumference), obesity (assessed by BMI) and an elevated blood pressure recording at the WPHC cross-sectional survey. By studying a sample of the Indigenous populations of Australia, which have some of the highest prevalence of hypertensive pregnancy, we have emphasised pre-pregnancy obesity and hypertension as independent contributors to this disease. Both of these risk factors are potentially amenable to intervention before pregnancy.

Our results add to previous Australian research that has identified elevated pre-pregnancy BMI as an important factor in the development of serious hypertensive disorders of pregnancy [27]. A systematic review of international controlled studies of risk factors for preeclampsia at antenatal booking showed that raised BMI before pregnancy (relative risk 2.47; 1.66-3.67) is one of the few amenable to intervention [28]. Other factors known to increase risk that cannot be altered prior to pregnancy include previous history or family history of preeclampsia, multiple pregnancy, nulliparity, and maternal age among multiparous women. Our study highlights the need to focus health promotion efforts on encouraging healthy body weight among Aboriginal and Torres Strait Islander women of childbearing age.

Elevated pre-pregnancy blood pressure in this study was an additional predictor of a hypertensive disorder of pregnancy. Hypertension among Indigenous women and men aged 25–50 years has previously been shown to be highly prevalent in communities included in the study [12]. Pre-pregnancy blood pressure assessment in this high risk population is important because undiagnosed chronic hypertension can be masked in early pregnancy due to physiological changes that may cause initial decreases in blood pressure, then misdiagnosed later in the pregnancy as a gestational disorder when abnormal readings re-surface [1]. One of the best predictors of chronic hypertension for Aboriginal and Torres Strait Islander Australians is overweight and obesity established by elevated waist circumference [17].

Aside from pre-pregnancy adiposity and raised blood pressure, two additional risk factors associated with poor metabolic health, elevated albumin:creatinine and the γ -glutamyl transferase, increased the risk for hypertension during pregnancy among the cohort. Raised albumin:creatine ratio as a marker of kidney damage and raised γ -glytamyl transferase as a marker of non-alcoholic fatty liver disease or risky alcohol use, provide a general picture of poor health in women of childbearing age. Among women in the cohort free of diabetes at baseline, presence of metabolic syndrome as defined by the International Diabetes Federation criteria, showed over a three-fold risk for hypertension during pregnancy. From a population health perspective, health risks among this cohort are exacerbated by high rates of smoking and risky alcohol use and low levels of physical activity. The extent of health challenges faced by these young women, and our improved understanding of how ‘fetal programming’ predicts the immediate and long term health of the next generation, call for interventions well before pregnancy begins in this population.

Encouraging weight loss during pregnancy is not a suitable strategy; weight loss outside of pregnancy requires lowering caloric intake or raising energy expended. Programs that incorporate a combination of physical activity and nutrition education have been shown to reduce body weight [29] and blood pressure [30,31]. In this study, less than 20% of the women reported undertaking adequate physical activity in the week before their baseline health check. It has been shown that when women exercise between 3 months and 1 year before or during pregnancy, the risk of preeclampsia is reduced [32,33]. The benefits of exercise prior to pregnancy for reducing the risk of preeclampsia, suggest that weight reduction interventions should feature well-designed physical activity components that focus on fitness, nutrition and weight loss in preference to weight loss alone.

This study is limited by its small sample size and low participation rate of eligible women (<50%) in the baseline survey and there is a possible downward and upward response bias to self-reported variables including current smoking status, alcohol use, fruit and vegetable intake and levels of physical activity. Despite the low participation rate, the cohort was well characterised at baseline with standardised body and biochemical measurements and the internal validity of the associations found between baseline risk and later hypertension in pregnancy should be high. A potential limitation of the study is the ability of perinatal data collections to report maternal health indicators accurately. A Canadian validation study found that gestational hypertensive disorders were more accurately coded (sensitivity 88%) than other variables, for example fetal/birth asphyxia (sensitivity 14.3%) [34]. The study is further limited by lack of data regarding whether or not women had a hypertensive disorder during a previous pregnancy as risk factors for recurrence are likely to be different for those women whose pregnancy is affected for the first time. The use of measured, rather than self-reported, anthropometric variables is an advantage in the study particularly because of the importance of body mass and central adiposity in predicting hypertension in pregnancy.

## Conclusions

Our study findings have enhanced our knowledge base, in general, about risk for hypertension in pregnancy. These data demonstrate high levels of obesity and poor metabolic health among Aboriginal and Torres Strait Islander women of child-bearing age in north Queensland that increase the risk for hypertensive disorders of pregnancy. Levels of physical activity were low. The study was undertaken in remote communities situated in isolated areas of Cape York and on Torres Strait Islands that frequently have limited resources for access to secure supplies of healthy foods and safe exercise environments. As well as improving food supply, consideration should be given to improving community infrastructures to include identified facilities (gymnasiums, walking paths and improved night lighting) which provide physical activity programs aimed at improving the health of young women prior to their first pregnancy and between pregnancies. Beneficial effects of better metabolic health of a woman of childbearing age may also have a strong positive effect on the health of her children across their lifespan.

## Competing interests

The authors declare that they have no competing interests.

## Authors’ contributions

The contributions of each author in this work are SC performed the data analysis, interpreted the data and drafted the manuscript. RM conceived and implemented the baseline study and linked hospitalisations data. JL, AE and RM contributed to data analysis and critical revision of the manuscript. All authors read and approved the final manuscript.

## Pre-publication history

The pre-publication history for this paper can be accessed here:

http://www.biomedcentral.com/1471-2458/13/138/prepub
